# Serum-free culture with PVP enables maintenance of primary culture of parathyroid cells *in vitro*

**DOI:** 10.1042/BSR20253933

**Published:** 2026-05-29

**Authors:** Natsumi Iwawaki, Toshiyuki Takehara, Atsuhiro Tatemizo, Takeshi Teramura

**Affiliations:** 1 Division of Cell Biology for Regenerative Medicine, Institute of Advanced Clinical Medicine, Kindai University Hospital, Sakai, Osaka, Japan; 2 Life Science Institute, Kindai University, Sakai, Osaka, Japan

**Keywords:** Epithelial-to-mesenchymal transition, Parathyroid cells, Polyvinylpyrrolidone, Serum-free culture

## Abstract

The parathyroid gland is a critical endocrine organ responsible for maintaining serum calcium homeostasis through the secretion of parathyroid hormone (Pth). Parathyroid tissue can be irreversibly lost due to autoimmune diseases or surgical injury, and the absence of appropriate model cells has hindered drug discovery targeting this cell type. Therefore, there is a strong need to develop *in vitro* culture systems for normal parathyroid cells for both cell therapy and pharmacological research. In the present study, we identified a serum-free culture system supplemented with B27 and polyvinylpyrrolidone that enables the maintenance of parathyroid-like cells. These cells retained expression of key markers such as calcium-sensing receptor and Pth and exhibited slow but consistent colony formation and proliferation. Notably, the cultured cells were highly sensitive to serum exposure; the addition of 10% fetal bovine serum rapidly induced epithelial-to-mesenchymal transition-like changes and a marked loss of functional gene expression. These findings not only provide a foundational strategy for culturing parathyroid cells but also highlight their inherent vulnerability to phenotypic and functional deterioration.

## Introduction

The parathyroid gland is a small endocrine organ located on the dorsal side of the thyroid gland. Despite its size, it plays a critical role in maintaining systemic homeostasis by secreting parathyroid hormone (PTH), which regulates blood calcium (Ca^2+^) levels [1]. Parathyroid cells sense extracellular Ca^2+^ concentrations through calcium-sensing receptor (CaSR) and adjust PTH secretion accordingly. This regulation controls calcium metabolism via bone resorption, renal tubular reabsorption, and intestinal absorption through vitamin D activation [2]. Dysfunction of parathyroid cells lead to disorders such as primary hyperparathyroidism and hypoparathyroidism, causing acute symptoms including tetany, seizures, psychiatric disturbances, and cardiac arrhythmias due to hypocalcemia, as well as chronic complications such as nephrocalcinosis and ectopic calcification [3,4]. Current treatments primarily rely on calcium supplements and active vitamin D analogs, which cannot replicate physiological PTH secretion, often resulting in fluctuating calcium levels and increased risk of renal impairment [5,6]. As a result, regenerative therapies involving cell transplantation to restore physiological PTH secretion have attracted growing attention.

Although autologous transplantation of parathyroid tissue is clinically performed, tissue availability is limited [7,8], and reliable *in vitro* expansion of normal parathyroid cells remains challenging. Early studies demonstrated that serum-containing conditions rapidly reduce CaSR expression and calcium responsiveness [9,10]. Serum-free systems partially preserve function; however, long-term phenotypic stability and suppression of fibroblast overgrowth remain unresolved issues [11–14]. Notably, three-dimensional (3D) culture systems, including collagen-based matrices and organoid approaches, have enabled temporary preservation of calcium responsiveness and PTH secretion, underscoring the importance of maintaining aspects of the native microenvironment for parathyroid cell stability [15–18]. Nevertheless, a simple and reproducible two-dimensional (2D) culture system capable of sustaining parathyroid cell identity under defined conditions has not been established.

Macromolecular crowding (MMC) has been proposed as a strategy to modify the pericellular environment *in vitro* [19,20]. In physiological tissues, the extracellular space is densely occupied by macromolecules, which influence diffusion dynamics and accumulation of secreted components. Polyvinylpyrrolidone (PVP), a high-molecular-weight polymer, has been used to induce MMC and has been reported to enhance extracellular matrix (ECM) deposition and alter the extracellular milieu in defined culture systems [21]. Incorporating such crowding conditions may help to recreate aspects of the *in vivo* microenvironment within a two-dimensional culture system.

We demonstrate here that the early loss of parathyroid cell function during primary culture may be attributed to epithelial-to-mesenchymal transition (EMT)-like changes induced by serum stimulation. By replacing serum with a serum-free medium and supplementing it with the MMC agent PVP, we successfully achieved 14-day *in vitro* maintenance of parathyroid cells characterized by sustained CaSR expression and colony-forming capacity.

## Material and methods

### Animals

All animal experiments were conducted at the Animal Experimental Facility of Kindai University in accordance with institutional regulations and the ARRIVE guidelines. Experimental protocols were approved by the Institutional Animal Care and Use Committee of Kindai University (approval no. KAME-2023-002).

Mice were anesthetized with 3% isoflurane via vaporized inhalation for tissue collection. For euthanasia, animals were sacrificed by exsanguination following deep anesthesia with isoflurane.

### Primary culture of mouse parathyroid cells

Parathyroid tissues were collected under a microscope from the pharyngeal region of C57BL/6J mice (CLEA Japan Inc., Tokyo, Japan). After mechanical dissociation with a scalpel, the tissue fragments were subjected to enzymatic digestion with 0.2% collagenase type II (Wako, Tokyo, Japan) at 37°C for 30 min in an incubator. To ensure consistent and reproducible digestion conditions, the samples were continuously mixed using a tube rotator (Ogawa Seiki Co., Ltd, Tokyo, Japan) at a rotation speed of 25 rpm. Following enzymatic digestion, the samples were centrifuged, and the pellet was resuspended and seeded into a 96-well culture plate (one well per mouse; a pair of parathyroid glands).

The culture medium was prepared by mixing Dulbecco’s modified Eagle medium (DMEM) calcium-free (Nacalai Tesque, Kyoto, Japan) and Ham’s F-12 Nutrient Mix (Thermo Fisher Scientific, Waltham, MA, U.S.A.) at a 1:1 ratio, supplemented with CaCl_2_ (Wako) to a final calcium concentration of either 0.5 mM, 1.2 mM, or 3.0 mM. Fetal bovine serum (FBS) (Nichirei, Tokyo, Japan) was added at 10%, and B27 Supplement (Thermo Fisher Scientific) was added at 1× final concentration. To mimic the physiological MMC environment of the cytoplasm and serum, PVP (K-90; MW 360 kDa; Nacalai Tesque, Japan; Cat# 10208-32, Lot# M2A5766) was dissolved in the culture medium at 11.34 mg/ml by stirring at room temperature to achieve a fluid volume occupancy (FVO) of 54%. For sterility and to maintain molecular integrity, the solution was sterilized via a 0.22-μm filter before use. This concentration corresponds to the estimated FVO observed in cellular and serum environments and has been shown to effectively mimic MMC conditions [21].

Cells were incubated at 37°C in a humidified atmosphere containing 5% CO_2_, and the medium was replaced every other day.

### Quantitative RT-PCR

Total RNA was isolated using TRI Reagent^®^ (Molecular Research Center Inc., OH, U.S.A.) following the manufacturer’s protocol. The concentration and purity of the extracted RNA were assessed using a NanoDrop spectrophotometer (Thermo Fisher Scientific). RNA purity was verified by the A260/280 and A260/230 absorbance ratios, with only samples exhibiting an A260/280 ratio between 1.8 and 2.1 being used for subsequent experiments. Total RNA was reverse transcribed into cDNA using the PrimeScript RT Master Mix Kit (Takara Bio Inc., Shiga, Japan) according to the manufacturer's instructions. Quantitative PCR was performed using the TB Green Premix Ex Taq II (Tli RNaseH Plus) (Takara Bio Inc.) in accordance with the manufacturer’s protocol. PCRs were conducted in a Thermal Cycler Dice^®^ Real Time System Single at 95°C for 20 s, followed by 40 cycles of 95°C for 5 s and 60°C for 30 s. To quantify the relative expression of each gene, the Ct (threshold cycle) values were normalized to those of *Gapdh* or *Tbp* (ΔCt = Ct target – Ct *Gapdh* or Ct *Tbp*) and compared with a calibrator using the ΔΔCt method (ΔΔCt = ΔCt sample – ΔCt control). All experiments were performed using three independent biology replications (*n* = 3). Statistical significance was evaluated with JMP software version 17.0.0 (SAS Institute) using the Tukey–Kramer honestly significant difference (HSD) test. The primer sequences are listed in Supplementary Table S1.

### Immunofluorescence staining

For immunofluorescence staining, samples were fixed in 4% formaldehyde solution (Wako) for 30 min at RT, permeabilized in 0.2% Triton-X (Wako) PBS(-) (Thermo Fisher Scientific) for 10 min, and treated with 10% Block Ace (Dainippon Sumitomo Pharma, Osaka, Japan) for 1 h at room temperature. Then, the samples were washed twice with 0.1% Triton-X in PBS(-) and incubated with primary antibody diluted in 10% Block Ace-PBS(-) overnight at 4°C. The samples were washed twice with 0.1% Triton-X in PBS(-) and reacted with secondary antibody diluted in 10% Block Ace-PBS(-) for 1 h. Prior to fluorescence observation, the samples were counter-stained with DAPI (Vector Laboratories, CA, U.S.A.) according to the manufacturer’s instructions. Images were acquired using the confocal laser-scanning microscope FV3000 (Evident Scientific, Tokyo, Japan) and FV31S-SW (Evident Scientific). Antibodies and dilution conditions are described in Supplementary Table S2.

### Paraffin section and HE staining

Parathyroid tissue samples were fixed in 4% paraformaldehyde (Wako) at room temperature for 24 h. Following fixation, tissues were dehydrated through a graded ethanol series, cleared in G-NOX (Nihon genetics, Tokyo, Japan), a biodegradable xylene substitute, and embedded in paraffin (Sakura Finetech Japan, Tokyo, Japan). Paraffin-embedded tissues were sectioned at a thickness of 3 μm using a microtome (TU-213; Yamato Kohki Industrial Inc., Saitama, Japan) and mounted onto silane-coated glass slides (Matsunami Glass Ind., Ltd., Osaka, Japan). The sections were deparaffinized with G-NOX, rehydrated through a descending ethanol series, and subsequently stained with Mayer’s hematoxylin and eosin (Sakura Finetech Japan) in accordance with the manufacturer’s protocol. After staining, sections were dehydrated, cleared in G-NOX, and cover-slipped with EntellanR New mounting medium (FUJIFILM, Tokyo, Japan). Histological images were acquired using a bright-field microscope (NIKON ECLIPSE Ts2; Nikon Corporation, Tokyo, Japan). Data acquisition and analysis were performed using NIS-Element L (NIKON).

### Cell count

Cultured cells were fixed with 4% paraformaldehyde and stained with DAPI to visualize nuclei, according to the manufacturer’s protocol. Fluorescent images were acquired using a fluorescence microscope (BZ-X810; Keyence, Tokyo, Japan). Data acquisition and analysis were performed using BZ-X analyzer (Keyence). To ensure objectivity and reproducibility, the number of cells was quantified by counting DAPI-stained nuclei using ImageJ software (National Institutes of Health). Briefly, images were converted to 8-bit grayscale, and an identical threshold value was applied to all images within each experimental set using the Create Mask function. The Analyze Particles command was then utilized to automatically count the nuclei, assuming each nucleus corresponds to one cell. Quantification was performed using three independent biological replicates (*n* = 3). Statistical analysis was conducted using JMP software version 17.0.0 (SAS Institute, Cary, NC, U.S.A.). Data are presented as mean ± standard deviation (SD), and statistical significance was evaluated using the Tukey–Kramer HSD test.

For mouse parathyroid gland, the tissue fragments were subjected to enzymatic digestion with 0.2% collagenase at 37°C for 30 min with rotation. The pellet was resuspended in antibody solution and incubated at 4°C for 30 min with rotation. The samples were washed with DMEM (Thermo Fisher Scientific) and resuspended in FACS buffer (DMEM supplemented with 2% FBS and 2 mM HEPES). Parathyroid cell numbers were analyzed using the FACS Aria II (BD Biosciences, SJ, CA). Data acquisition and analysis were performed using FACS Diva software v8.1.1 (BD Biosciences). Antibodies and dilution conditions are described in Supplementary Materials.

### Statistical analysis

All data are presented as the mean ± SD. The number of independent biological replicates (n) for each experiment is indicated in the corresponding figure legends (*n* = 3 per group). For comparisons between two groups, the Student’s t-test was employed. For comparisons among three or more groups, one-way analysis of variance (ANOVA) followed by the Tukey–Kramer HSD post-hoc test was performed to determine specific differences between groups. All statistical analyses were conducted using JMP software version 17.0.0 (SAS Institute, Cary, NC, U.S.A.). A *P*-value <0.05 was considered statistically significant.

## Results

### Quantification of functional parathyroid cells in mouse glands

**Figure 1:**
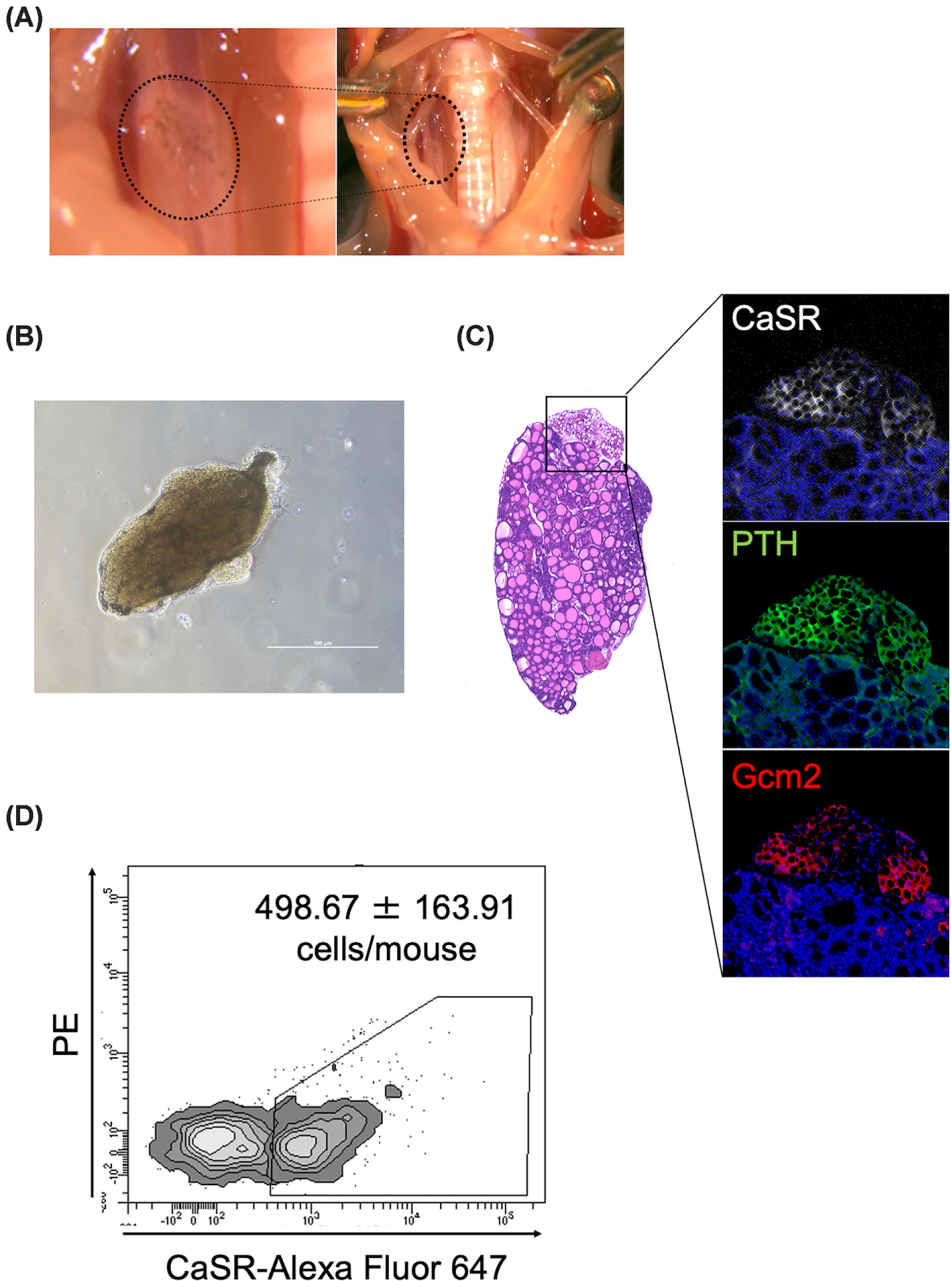
Isolation and quantification of functional parathyroid cells in mice (**A**) Anatomical location of the parathyroid glands in the cervical region of C57BL/6J mice. (**B**) Gross appearance of the resected tissue block containing parathyroid glands. (**C**) Histological and immunofluorescence staining of the tissue using H&E, anti-CaSR (white), anti-Gcm2 (red), and anti-PTH antibodies (green), with DAPI (blue) as nuclear counterstain. (**D**) Quantification of CaSR-expressing parathyroid cells in parathyroid gland using flow cytometry with Alexa Fluor647-conjugated anti-CaSR antibody. Data represent the mean ± SD from three independent experiments (*n* = 3 mice per group).

To establish a starting point for primary culture, we quantified the number of functional parathyroid cells in individual mice. The glands were identified as clusters on the dorsal side of the thyroid (Figure 1A,B). Immunofluorescent analysis confirmed that these cells express the parathyroid markers CaSR, PTH, and GCM2 (Figure 1C). Following enzymatic dissociation, the cell suspension was labeled with an Alexa Fluor 647-conjugated anti-CaSR antibody and analyzed by flow cytometry. The number of CaSR-positive cells was estimated to be 498.67 ± 163.91 per mouse (Figure 1D).

### Serum-free medium containing B27 and PVP maintains epithelial characteristics of parathyroid cells

**Figure 2:**
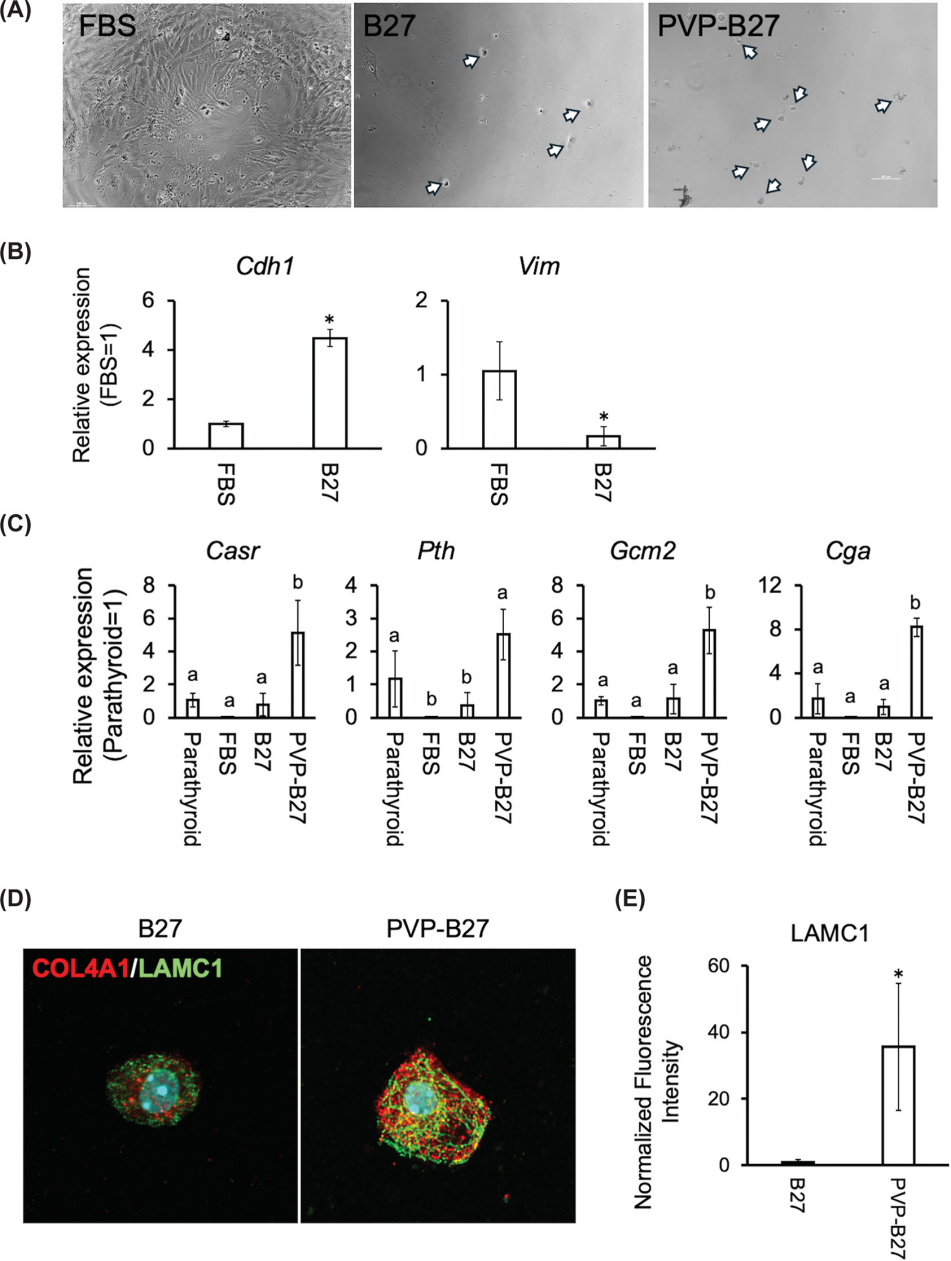
Emergence and epithelial characteristics of primary mouse parathyroid cells in serum-free medium supplemented with B27 and PVP (**A**) Representative images of parathyroid cells 14 days post-seeding in FBS, B27, and PVP-B27 media. Arrows indicate cells exhibiting epithelial-like morphology. (**B**) Relative expression levels of the epithelial marker *Cdh1* and the mesenchymal marker *Vim*, assessed by qPCR. Data are presented as mean ± SD from three biological replicates. Asterisks indicate statistically significant differences (*P* <0.05). (**C**) Expression of parathyroid-specific marker genes *Casr*, *Pth*, *Gcm2*, and *Cga* under the serum-free conditions. Data represent mean ± SD from three biological replicates. Different letters indicate statistically significant differences (*P* <0.05). (**D**) Representative immunofluorescence images of parathyroid primary cultured cells after 14 days in serum-free medium supplemented with B27 and B27-PVP. The cells were stained for LAMC1 (red) and COL4A1 (green), with DAPI (blue) as a nuclear counterstain. (**E**) The fluorescence intensities of LAMC1 were quantified from three images per experiment using ImageJ software. Data are presented as mean ± SD (*n* = 3 independent biological replicates). Different letters indicate statistically significant differences (*P* <0.05) determined by one-way ANOVA followed by the Tukey–Kramer HSD test.

In conventional *in vitro* culture systems, the expression of key parathyroid markers *Casr* and *Pth* typically declines. We therefore investigated whether serum-free conditions could preserve the gene expression associated with parathyroid function. After 14 days in culture, cells in conventional serum-containing medium predominantly exhibited fibroblast-like morphology without the formation of epithelial colonies (Figure 2A). In contrast, cultures in serum-free medium supplemented with B27 supported the emergence of cells with epithelial-like morphology and maintained expression of the epithelial marker *Cdh1*, while suppressing the mesenchymal marker *Vim* (Figure 2A,B).

To further enhance functional maintenance, we examined the effect of adding PVP as a MMC agent. Supplementation with PVP improved cell viability after 14 days (Figure 2A and Supplementary data 1A). Notably, parathyroid marker genes including *Casr, Pth, Gcm2*, and *Cga* were significantly up-regulated in cultures maintained in serum-free medium supplemented with PVP compared with B27 alone (Figure 2C and Supplementary data 1B).

To assess whether PVP-mediated MMC was associated with ECM remodeling, we examined the expression and deposition of basement membrane–related ECM components. Quantitative RT-PCR revealed that Lamc1 and Col4a1 mRNA levels were significantly higher in PVP-B27 cultures than in B27 alone (Supplementary Data 2A). Immunofluorescence staining revealed increased LAMC1 and COL4A1 signals in PVP-treated cultures compared with B27 alone, with prominent pericellular staining patterns (Figure 2D and Supplementary Data 2B). Consistently, quantitative image analysis demonstrated a significant increase in LAMC1 fluorescence intensity in the presence of PVP (Figure 2E).

### Calcium supplementation promotes proliferation of parathyroid cells

**Figure 3:**
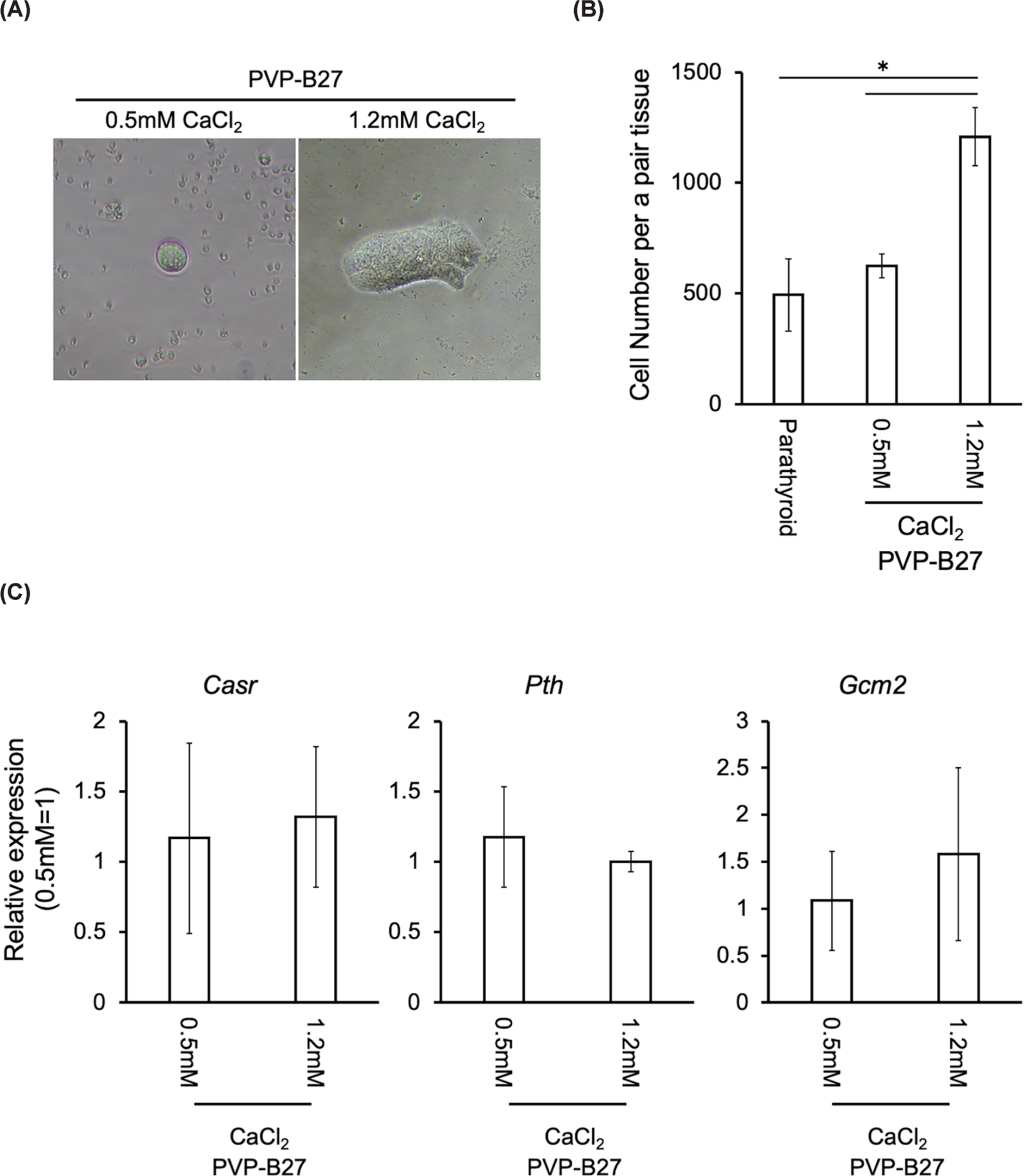
Effect of 1.2 mM calcium supplementation on the phenotype and proliferation of parathyroid cells (**A**) Colony formation by parathyroid cells cultured under serum-free conditions with 1.2 mM Ca^2+^. (**B**) Calcium supplementation promotes parathyroid cell proliferation. Data represent mean ± SD from three independent biological replicates. Asterisks indicate statistically significant differences (*P* <0.05). (**C**) Relative expression of parathyroid-specific marker genes *Casr*, *Pth*, and *Gcm2* under calcium-supplemented conditions. Data represent mean ± SD from three independent biological replicates. Asterisks indicate statistically significant differences (*P* <0.05).

While PVP-supplemented medium stabilized the phenotype, this condition alone was insufficient to induce robust *ex vivo* expansion. Therefore, we examined the dose-dependent effect of extracellular calcium concentration (0.5 and 1.2 mM) on cell growth. Cell numbers increased in a calcium concentration-dependent manner. Under 1.2 mM calcium, cell numbers (1207.67 ± 131.36, *n* = 3) were significantly higher than those under 0.5 mM (625.33 ± 53.93, *n* = 3) and parathyroid tissue at isolation (493.67 ± 163.54, *n* = 3) (*P* <0.05), representing approximately a 2.4-fold increase compared with the parathyroid tissue at isolation (Figure 3A,B).

In contrast, a higher concentration of 3.0 mM resulted in cell death (Supplementry data 3). These findings indicate that a calcium concentration of 1.2 mM supports efficient *ex vivo* expansion of parathyroid-like cells in this system. Importantly, the proliferating cells maintained expression of parathyroid-specific marker genes (Figure 3C).

### Serum stimulation induces EMT and rapid functional loss in parathyroid cells

**Figure 4:**
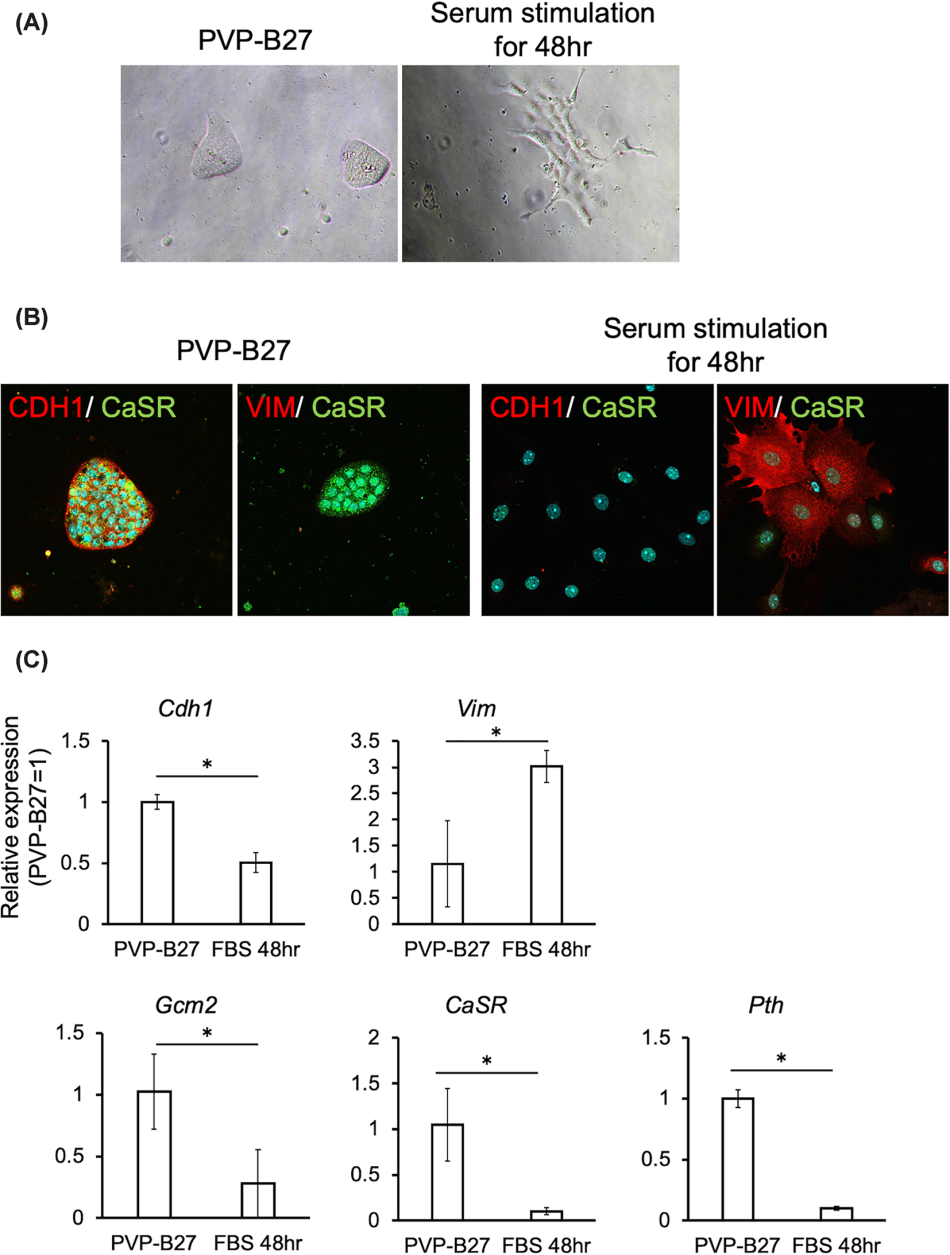
Serum exposure induces EMT-like changes and loss of function in cultured parathyroid cells (**A**) Morphological changes from epithelial-like to fibroblastic appearance after 48-h serum exposure. (**B**) Immunofluorescence staining for CaSR (parathyroid marker), CDH1 (CADHERIN1; epithelial marker), and VIM (VIMENTIN; mesenchymal marker) in parathyroid cells cultured with or without serum for 48 h. (**C**) Gene expression analysis of parathyroid markers *Gcm2*, *CaSR*, and *Pth*; epithelial marker *Cdh1*; and mesenchymal marker *Vim* before and after serum exposure. Data are presented as mean ± SD from three independent biological replicates. Asterisks indicate statistically significant differences (*P* <0.05).

We investigated the factors contributing to the loss of parathyroid identity by examining the impact of serum exposure on cells previously maintained under serum-free conditions. Upon transfer to serum-containing medium, the cells rapidly transitioned from an epithelial to a fibroblast-like morphology within 48 h (Figure 4A). Immunostaining and gene expression analyses revealed a marked up-regulation of the mesenchymal marker Vimentin and a concomitant decrease in the epithelial marker *Cdh1* (Figure 4B). Concurrently, the expression of key parathyroid markers *Casr, Pth*, and *Gcm2* was significantly down-regulated (Figure 4C). These results demonstrate that serum exposure induces a rapid loss of parathyroid cell identity and function.

## Discussion

Parathyroid cells are highly specialized endocrine epithelial cells that rapidly secrete PTH in response to subtle fluctuations in extracellular calcium. This function depends on preserved cell polarity, stable cell–cell adhesion, and a highly differentiated state. Numerous methods have been explored for culturing normal parathyroid cells *in vitro*; however, many studies have reported that conventional culture conditions are rapidly compromised by fibroblast overgrowth and the early loss of parathyroid-specific characteristics, such as the expression of *Casr* and *Pth* [22,23]. As a result, long-term maintenance of parathyroid cells in 2D culture has proven to be challenging. More recently, a 3D organoid culture using collagen matrices was shown to maintain calcium responsiveness and PTH secretion for up to two weeks, though fibroblast expansion eventually compromised long-term culture integrity. These studies highlight two key notions: serum-free conditions better preserve function, and fibroblast overgrowth must be suppressed for stable culture.

Our findings demonstrated that serum supplementation hinders the selective expansion and functional maintenance of parathyroid cells. In conventional serum-containing medium, fibroblast-like cells predominated, exhibiting low expression of parathyroid markers (*Casr, Pth, Gcm2*) and the epithelial marker *Cdh1*, alongside increased mesenchymal marker *Vim*. While serum-free conditions suppressed the emergence of fibroblastic cells, the resulting cells initially showed unstable expression of parathyroid-specific genes. Although their marker expression was slightly higher than in serum-supplemented conditions, it remained comparable to native parathyroid tissue, which includes many stromal cells, suggesting incomplete functional retention. Chemically defined serum-free media often lack soluble ECM proteins and cytokines, which are crucial for cell proliferation, survival, and functional maintenance [24]. To compensate for this, we introduced PVP as a MMC agent to modify the extracellular diffusion environment. Under PVP-based MMC conditions, we observed a marked stabilization of parathyroid and epithelial marker expression. Beyond passive molecular confinement, MMC was associated with increased Lamc1 and Col4a1 mRNA levels and enhanced ECM deposition, suggesting reinforcement of the ECM at both transcriptional and deposition levels. Such ECM enrichment may contribute to the stabilization of epithelial architecture [25]. In parallel, Casr expression was preserved at both mRNA and protein levels under MMC conditions, suggesting that maintenance of parathyroid identity may be linked to an ECM-supported microenvironment.

The regulation of extracellular calcium is equally critical, as it activates CaSR to suppress both PTH secretion and cell proliferation [26]. Consequently, precise calcium control is indispensable for *in vitro* culture. While early studies suggested that low-calcium conditions (0.3–0.5 mM) support parathyroid growth [11,23], recent evidence indicates that prolonged hypocalcemia can lead to hypertrophy, growth arrest, and structural abnormalities, such as dilation of the rough ER [27–29]. In contrast, high-calcium conditions (2.0 mM) or CaSR agonists strongly activate CaSR signaling, which inhibits proliferation through cell cycle regulators like the MAPK/p21/p27 pathway [30]. Notably, intensive CaSR activation has been shown to induce the regression of hyperplastic parathyroid glands through accelerated apoptosis [31,32]. In our study, we observed significant proliferation at 1.2 mM calcium, a concentration close to the murine calcium set point (1.0–1.2 mM). This suggests that proliferation at physiological levels may be associated with partial CaSR activation. Conversely, at 3.0 mM calcium, mimicking severe hypercalcemia, we observed extensive cell death. This outcome likely reflects the CaSR-mediated pro-apoptotic response observed *in vivo*, suggesting that our *in vitro* model may recapitulate aspects of the dynamic shifts between cell proliferation and apoptosis.

Furthermore, our results confirmed that serum exposure induces EMT-like changes, including the up-regulation of *Vim* and down-regulation of *Casr, Pth*, and *Gcm2*. Such EMT-driven functional loss is also reported in other secretory epithelial cells like pancreatic beta-cells [33,34], thyroid follicular cells [35], and airway epithelial cells [36], where disruption of polarity can lead to decline in function. Serum contains growth factors such as TGF-β, FGF, and IL-6 [37–39], which may contribute to mesenchymal-like transition observed under serum-containing conditions. These findings indicate that parathyroid cells are highly sensitive to EMT, and minimizing serum exposure is crucial for preserving their identity.

A primary limitation of the present study is the lack of PTH protein quantification. In the murine model, parathyroid glands yield only approximately 500 cells per individual, which was insufficient to reach the detection threshold of standard ELISA. Consequently, our characterization was primarily based on the sustained mRNA expression of *Casr* and *Pth*. Furthermore, the long-term functional stability of these cells after *in vivo* transplantation remains to be validated. Future studies utilizing larger animal models, such as bovine or porcine, will be instrumental in overcoming these cell-number constraints to robustly quantify protein secretion and assess the translational potential of this culture system.

In conclusion, we propose a 2D serum-free culture method that supports the maintenance of parathyroid identity under PVP-mediated MMC conditions. While organoid-based approaches have advanced endocrine tissue engineering, our 2D system offers a simple, reproducible, and scalable alternative. This platform is well-suited for drug screening and disease modeling, where rapid and reliable *in vitro* assessment of hormone secretion and stress responses is required.

## Supplementary Material

Supplementary Figures S1-S3 and Tables S1-S2

## Data Availability

The data that support the findings of the present study are available from the corresponding author upon reasonable request.

## Open Access

Open access for this article was enabled by the participation of Kindai University in an all-inclusive Read & Publish agreement with Portland Press and the Biochemical Society.

## Abbreviations

CaSR: calcium-sensing receptor
DMEM: Dulbecco’s modified Eagle medium
EMT: epithelial-to-mesenchymal transition
ECM: extracellular matrix
FBS: fetal bovine serum
FVO: fluid volume occupancy
HSD: honestly significant difference
MMC: Macromolecular crowding
PTH: parathyroid hormone
PVP: polyvinylpyrrolidone
SD: standard deviation
