# Supplementary material for: Serum-free culture with PVP enables maintenance of primary culture of parathyroid cells in vitro
Source: Biosci Rep. 2026 May 29;46(6):BSR20253933. doi: 10.1042/BSR20253933 (PMC13223393; doi:10.1042/BSR20253933)
Supplement: Supplementary Figures S1-S3 and Tables S1-S2 [file BSR-2025-3933_supp.pdf]

Supplementary data 3.

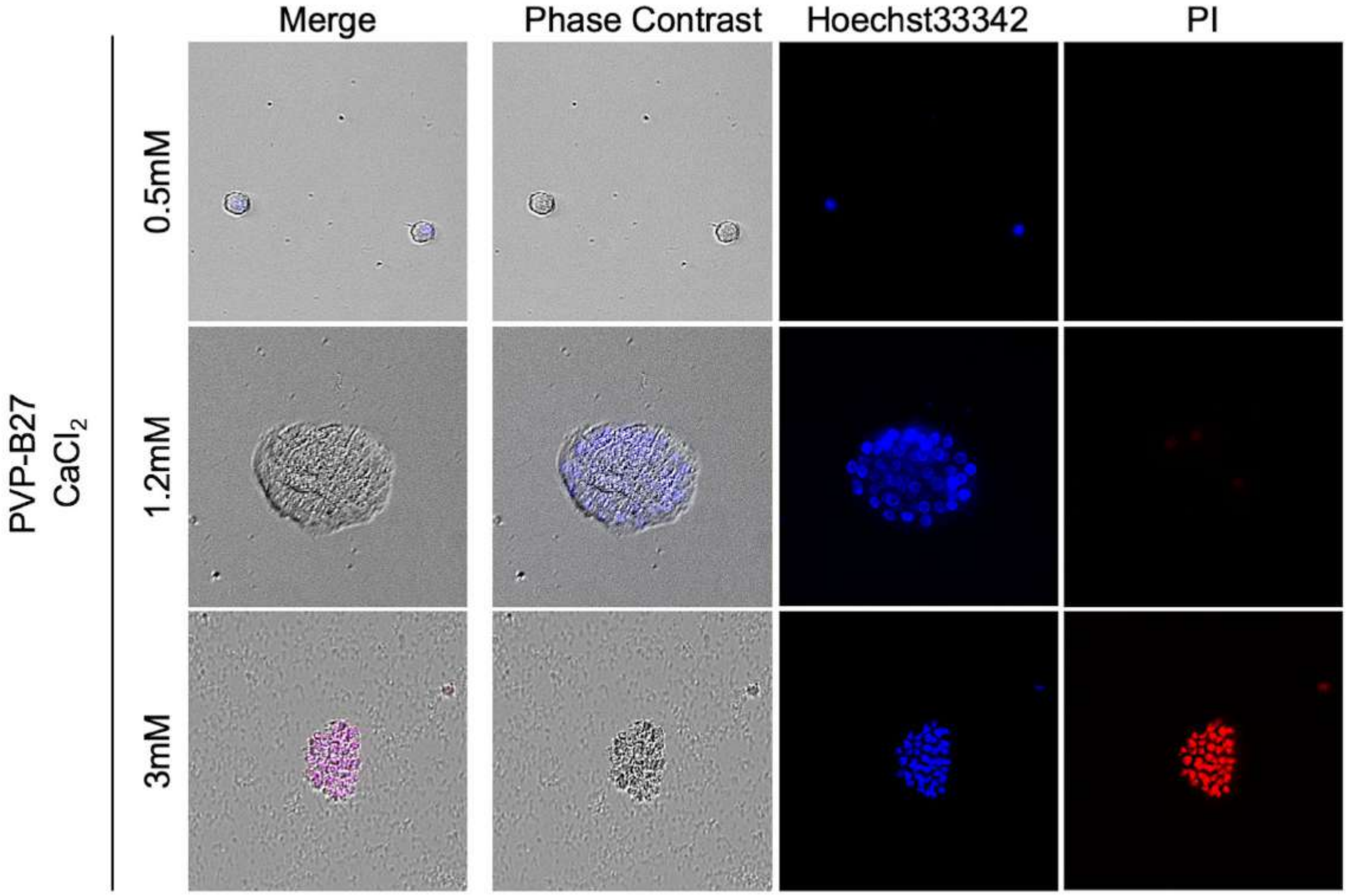

Supplementary data 3. Effects of calcium concentration in PVP-B27 serum free medium on parathyroid cell viability.

Primary mouse parathyroid cells were cultured under PVP-B27 with $CaCl_2$ at 0.5, 1.2, or 3.0 mM for 14 days. Representative phase-contrast and fluorescence images are shown. Nuclei were stained with Hoechst33342 (blue), and dead cells were identified by propidium iodide (PI; red).

Supplementary data 2.

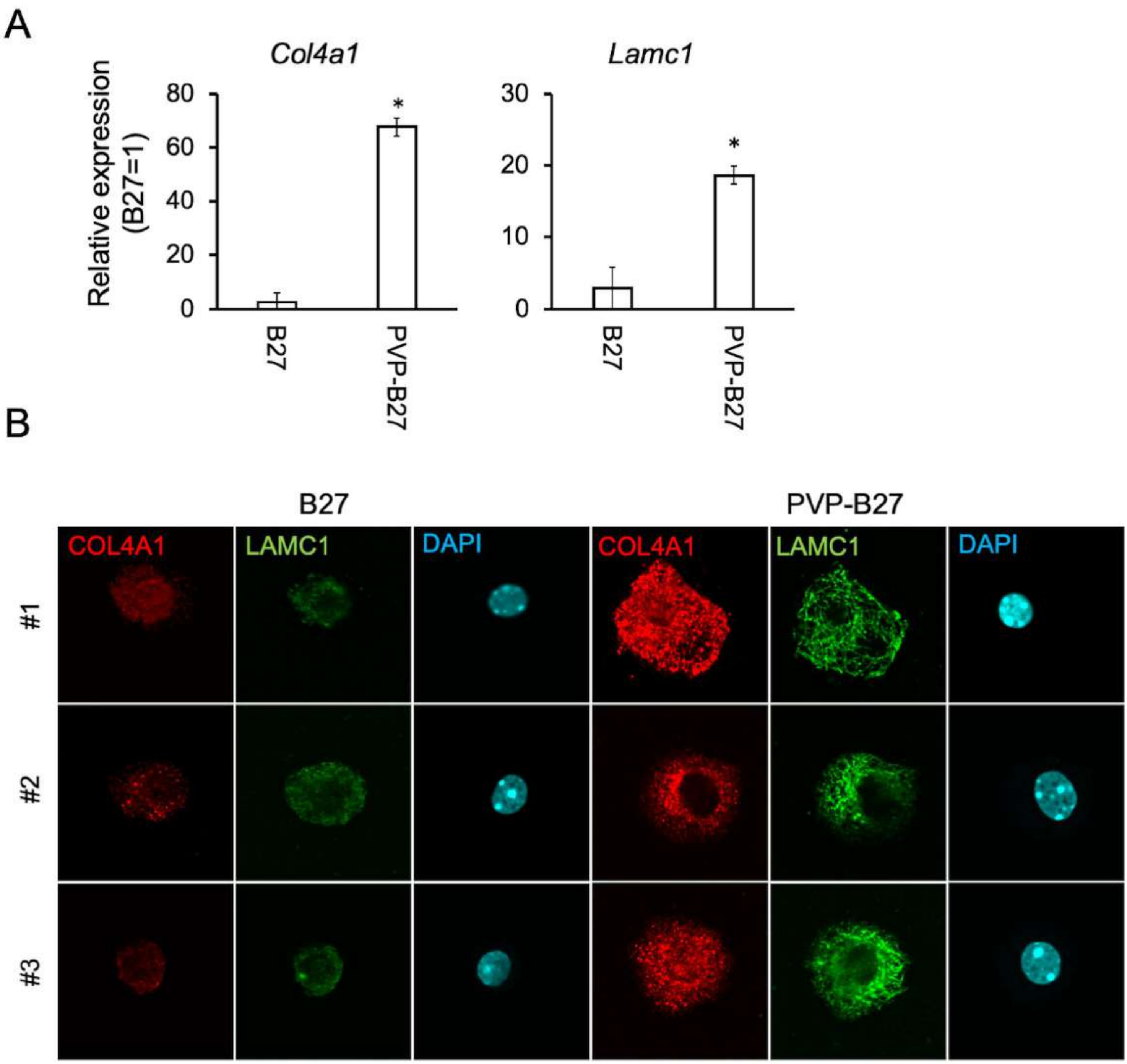

Supplementary data 2. PVP-mediated macromolecular crowding enhances ECM expression and deposition.
(A) Quantitative RT-PCR analysis of *Col4a1* and *Lamc1* mRNA expression. Parathyroid cells were cultured in B27 or PVP-B27 medium. Relative expression levels of *Col4a1* and *Lamc1*, assessed by qPCR. Data are presented as mean ± SD from three independent biological replicates. Asterisks indicate statistically significant differences ($P < 0.05$). (B) Immunofluorescence staining for COL4A1 and LAMC1. Representative images show the deposition of COL4A1 (red) and LAMC1 (green) in parathyroid cells cultured under B27 (left) and PVP-B27 (right) conditions. Nuclei were counterstained with DAPI (blue). Note the marked increase in both intracellular localization and extracellular deposition of ECM components in the presence of PVP.

Supplementary data 1.

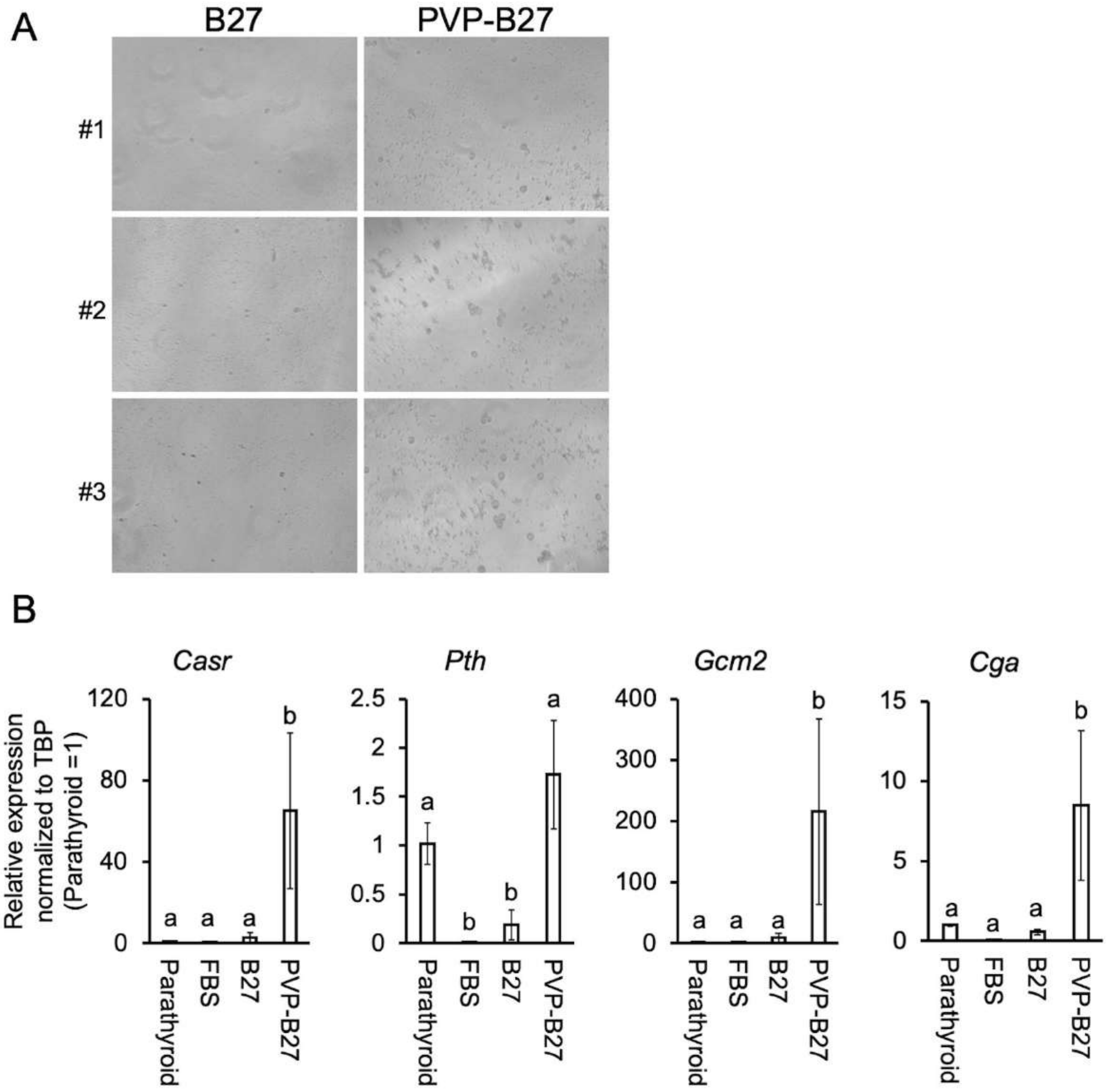

Supplementary data 1. Effect of PVP supplementation on colony formation and parathyroid-associated gene expression under serum-free conditions.

(A) Representative phase-contrast images of primary mouse parathyroid cells cultured under serum-free conditions supplemented with B27 alone (B27) or with PVP and B27 (PVP-B27). Images from three independent biological replications (#1–#3) are shown. Increased cell viability was observed under PVP-B27 conditions compared with B27 alone. (B) Relative mRNA expression levels of *Casr*, *Pth*, *Gcm2*, and *Cga* analyzed by quantitative RT-PCR. Expression levels were normalized to *Tbp* and are shown relative to isolated parathyroid gland (Parathyroid = 1). Cells were cultured under the indicated conditions (FBS, B27 and PVP-B27). Data are presented as mean ± SEM (n = 3 biological replicates). Different letters indicate statistically significant differences between groups ($P < 0.05$, one-way ANOVA followed by Tukey-Kramer HSD test).

Table S1. Primer list used in this study.

	Primer sequence (5' to 3')	
Gene	Forward	Reverse
Cdh1	TCTGTCGCCACTTTGAATC	ATGTCCTGGGCAGAGTGAG
Vim	GCCGAGGAATGGTACAAGTC	GGCATCGTTGTTCCGGTT
Casr	AGTAGCAGCCAAAGATCAAG	TCTATGGCAAAGATCATGG
Gcm2	AACTTCTGGAGACTTGATGG	TCTTCTGCCTTCTGTCTCTG
Pth	TCATGCTGGCAGTCTGTC	TGTTTGCCCAGGTTGTG
Cga	ATGACAAAAGGGGACACC	ATCCTCTCGTCTCCTTGG
Tbp	AATGGTGTGCACAGGAG	AAGCCCAACTTCTGCAC
Gapdh	TCGTGGAGTCTACTGGTGTC	TCGTGGTTCACACCCATCAC

Table S2. Antibodies and brief conditions for the experiments.

Application	Antibody (Primary antibody)	Company	Cat No (Clone No)	Dilution	Condition
IF	Mouse monoclonal anti-CaSR Antibody	Abcam	ab19347(5C10, ADD)	1/100 in 10%Block-Ace/PBS	4°C, Overnight
IF	Rabbit Polyclonal anti-Vimentin Antibody	Proteintech	10366-1-AP	1/100 in 10%Block-Ace/PBS	4°C, Overnight
IF	Mouse anti-PTH Antibody	Biorad	7170-6216	1/100 in 10%Block-Ace/PBS	4°C, Overnight
IF	Rabbit polyclonal anti-Gcm2 antibody	Proteintech	PGI-21088-1-AP-150	1/100 in 10%Block-Ace/PBS	4°C, Overnight
FCM/IF	Mouse monoclonal anti-CaSR Alexa Fulor647	Santacruz	sc-47741 (6D4)	1/200 in DMEM/1/100 in 10%Block-Ace/PBS	4°C, 30min/4°C, Overnight

Application	Antibody (Secondary antibody)	Company	Cat No (Clone No)	Dilution	Condition
IF	Donkey Anti-IgG(H+L), Rabbit CFTM555	BTI	20038-1MG	1/1000 in 10% Block-Ace/PBS	RT, 1h
IF	Donkey Anti-IgG(H+L), Mouse CFTM488	BTI	20014-1MG	1/1000 in 10% Block-Ace/PBS	RT, 1h
